# Short-term associations between Legionnaires' disease incidence and meteorological variables in Belgium, 2011–2019

**DOI:** 10.1017/S0950268820000886

**Published:** 2020-04-29

**Authors:** T. Braeye, F. Echahidi, A. Meghraoui, V. Laisnez, N. Hens

**Affiliations:** 1 Department of Public Health and Surveillance, Sciensano, Brussels, Belgium; 2 Interuniversity Institute for Biostatistics and Statistical Bioinformatics, Data Science Institute, Hasselt University, Hasselt, Belgium; 3 Department of Microbiology and Infection Control, National Reference Center for *Legionella pneumophila*, Universitair Ziekenhuis Brussel, Vrije Universiteit Brussel (VUB), Brussels, Belgium; 4 Department of Microbiology, National Reference Center for *Legionella pneumophila*, Université Libre de Bruxelles (ULB), Laboratoire Hospitalier Universitaire de Bruxelles (LHUB-ULB), Hôpital Erasme Brussels, Brussels, Belgium; 5 Agency for Care and Health, Infection Prevention and Control, Flemish Community, Schaerbeek, Belgium; 6 Centre for Health Economics Research & Modelling Infectious Diseases (CHERMID), Vaccine & Infectious Disease Institute, University of Antwerp, Antwerp, Belgium

**Keywords:** Case-crossover study, *Legionella pneumophila*, meteorology

## Abstract

The number of reported cases with Legionnaires' disease (LD) is increasing in Belgium. Previous studies have investigated the associations between LD incidence and meteorological factors, but the Belgian data remained unexplored. We investigated data collected between 2011 and 2019. Daily exposure data on temperature, relative humidity, precipitation and wind speed was obtained from the Royal Meteorological Institute for 29 weather stations. Case data were collected from the national reference centre and through mandatory notification. Daily case and exposure data were aggregated by province. We conducted a time-stratified case-crossover study. The ‘at risk’ period was defined as 10 to 2 days prior to disease onset. The corresponding days in the other study years were selected as referents. We fitted separate conditional Poisson models for each day in the ‘at risk’ period and a distributed lag non-linear model (DLNM) which fitted all data in one model. LD incidence showed a yearly peak in August and September. A total of 614 cases were included. Given seasonality, a sequence of precipitation, followed by high relative humidity and low wind speed showed a statistically significant association with the number of cases 6 to 4 days later. We discussed the advantages of DLNM in this context.

## Introduction

*Legionella* spp. were first described in 1977 [1]. It is a Gram-negative intracellular pathogen that can be transmitted to humans via inhalation of aerosols. It can cause legionellosis: Legionnaires' disease (LD) and Pontiac fever [2]. LD is a cause of community acquired pneumonia, but *Legionella* also causes nosocomial infections. Almost 96% of LD cases in Europe are caused by the species *Legionella pneumophila*. The majority of reported *L. pneumophila* cases are linked to serogroup 1 [3].

Several countries have reported an increase in LD incidence in recent years [4]. Because of known effects of meteorology on *Legionella* spp., researchers have investigated changing weather and weather patterns as a possible cause of the increase in LD incidence. Meteorological variables affect growth and presence in the environment [5,6]. Precipitation and higher temperatures, for example, increase the growth of *Legionella* and its supporting organisms (photosynthetic primary producers, e.g. algae and cyanobacteria) [7]. Although these effects have been established, their clinical significance is still under investigation. The presence of *Legionella* is a poor predictor of infections [8] and environmental sampling during outbreaks has delivered mixed results [9,10]. Epidemiological research has tried to link clinical significance, LD incidence, to meteorological variables measured in the preceding days and weeks.

This research on short-term associations has not delivered consistent results. Inconsistency is most remarkable for temperature: non-linear [11–13], negative [14,15] and positive [16–19] associations have been reported. Similarly, for atmospheric pressure non-linear [12,16], negative [15] and positive [20] associations have been reported. The reported associations with relative humidity [5,6,11,13,15–17,21] and precipitation [5,6,11,12,15–18,22,23] have always been positive. Relative humidity has however also been included in studies without resulting in significance associations [19,22]. Significant negative associations have been reported for wind speed [5,15,21]. In addition, studies have added atmospheric stagnation, vapour pressure and changes in local watershed, the area that catches rain and snow, to the analysis and found that these showed stronger associations with LD incidence than traditionally reported meteorological variables [19,20,24].

Analysis of the effect of transient exposures on the variation in LD incidence is necessarily complex and some of the conflicting results can be caused by differences in methodology. Three issues should be introduced: non-linearity, seasonality and autocorrelation. Non-linearity can cause both high and low temperature to be associated with an increase in LD incidence. When only linear effects are allowed in the analysis, any significant association will be unidirectional [14,15,18,19]. Studies that allowed for non-linear effects have either categorised the meteorological variables, included cubic splines [12] or quadratic transformations of the variables [11].

As seasonality observed in both the LD incidence and in meteorological trends could be an important confounder, most researchers have eliminated seasonal variation from their analysis. The case-crossover design has been a popular design [5,6,15,17,20,22] because it allows for the elimination of seasonality through referent selection. Different referent selection strategies have been applied in LD research, but it is unclear if they completely eliminated time-varying confounding. If seasonality remains, there is a probability to find positive associations between LD incidence and temperature whenever LD incidence peaks during warmer seasons.

For short-term associations, the ‘at risk’ period of interest typically includes several days and statistically significant associations can be obtained for each of these days. To investigate associations on several consecutive days, researchers have either fitted separate models by day, selected a specific day by variable or averaged over several days. The use of values obtained on different days for the same variable in a model is uncommon because of temporal autocorrelation. Different meteorological variables are likely also correlated on the same day and over days. This issue, known as multicollinearity is avoided in previous studies by building separate models for correlated variables or selecting only one of the correlated variables after a preliminary analysis [15,19], principal component analysis [11] or by including interactions in the model [6,12,13,16,18,20]. Interaction terms however will themselves be correlated with the main effects and require more statistical power. Adding to the complexity is that effect modification by quarter and year has been observed for some interactions [6]. A possible solution for temporal autocorrelation over different days is distributed lag non-linear models (DLNM). These models allow for the inclusion of trends over multiple days (lags) and non-linear effects through splines [25]. To our knowledge they have not been applied in research on LD.

Some of Belgium's neighbouring countries (the Netherlands [11,12], the UK [6,17,21] and Germany [12]) have analysed data on LD incidence and meteorological variables, but the Belgian data remain unexplored. The objective of this paper is to investigate the short-term association (2–10 days prior to diagnosis) between meteorological variables (temperature, relative humidity, precipitation and wind speed) and LD incidence in Belgium. We explored the added value of DLNM in combination with case-crossover designs to overcome difficulties inherent to this analysis.

## Methods

### Data on LD incidence

LD cases were obtained from the Belgian National Reference Center and regional mandatory notification system with a date of disease onset between 1 January 2011 and 31 August 2019 [26]. Data sources were combined and duplicates were removed. Cases with the same birthdate, same sex, same postal code and for who the date of onset was less than 30 days apart were considered duplicates. We excluded travel-related (domestic or international travel up to 14 days before diagnosis), nosocomial cases (admission in a healthcare facility up to 14 days before diagnosis) and cases linked to outbreaks. Both confirmed and probable cases of *Legionella* spp. were included in the analysis. The laboratory criteria for case confirmation were: (1) the isolation of *Legionella* spp. from respiratory secretions or any normally sterile site [27], (2) detection of *L. pneumophila* antigen in urine, (3) a significant rise in the specific antibody level to *L. pneumophila* serogroup 1 in paired serum samples. The laboratory criteria for a probable case were: (1) detection of *L. pneumophila* antigen in respiratory secretions or lung tissue e.g. by direct fluorescent-antibody staining using monoclonal-antibody derived reagents, (2) detection of *Legionella* spp. nucleic acid in respiratory secretions, lung tissue or any normally sterile site [28,29], (3) significant rise in specific antibody level to *L. pneumophila* other than serogroup 1 or other *Legionella* spp. in paired serum samples, single high level of specific antibody to *L. pneumophila* serogroup 1 in serum.

### Meteorological data

**Fig. 1.**
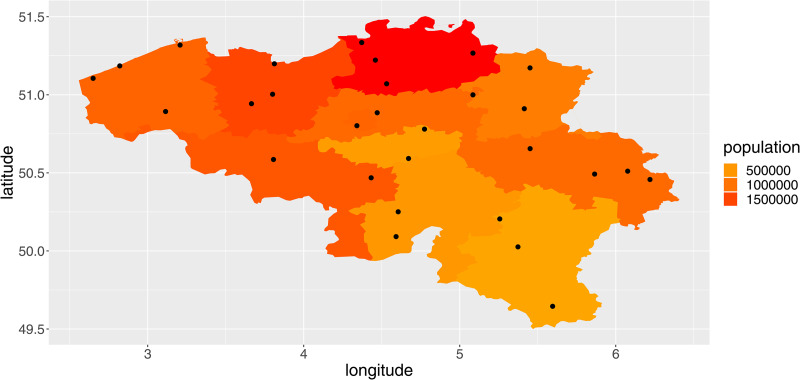
Overview of the provinces of Belgium, their population totals in 2018 and the location of the weather stations (red dots).

We included average daily temperature (°C), relative humidity (%), wind speed (m/s) and precipitation (mm) in the analysis. The data were obtained from the Royal Meteorological Institute of Belgium for all available weather stations (*N* = 29). Every province contained at least one weather station (Fig. 1).

### Data presentation

We fitted linear long-term trends to the meteorological variables. We described multicollinearity between meteorological variables by presenting the Pearson's correlation coefficient between daily national averages. We further presented seasonality and long-term trends in our LD-data by plotting the total and smoothed weekly national number of cases. We also plotted the smoothed and scaled meteorological variables. We scaled meteorological variables for presentation purposes by applying the linear transformation (*x* − mean(*x*))/S.D.(*x*).

### ‘At risk’ period

We defined our ‘at risk’ period as the period from 10 to 2 days before disease onset. This period corresponds to the incubation period [17,30].

### Case-crossover analysis

Daily case- and exposure-data were aggregated by province. We opted for a case-crossover design and fitted the data with conditional Poisson regression models. Referents were selected from the same province for the corresponding days in the eight other study years. Referent-selection eliminated day-to-day seasonality from the analysis. Two different models were used to fit the data. (1) Single-day models: fitting data for each day in the ‘at risk’ period with a separate model. In these models we included daily temperature, relative humidity and wind speed as factors after categorisation into quantiles. Precipitation was categorised in two categories (≤0.2 and >0.2 mm). (2) DLNM: fitting data from the entire ‘at risk’ period with one model. For a DLNM, we needed to create a cross-basis for each meteorological variable, which represent both the lag and the values of the meteorological variables. For the lag variable, we used a piecewise linear base. For the wind speed, precipitation and relative humidity variables, we used a linear base. We used a basic spline for temperature to allow for non-linearity. The reference values for presenting and predicting from the cross-bases were the median values of the meteorological variables over the study period.

All models contained the four meteorological variables, a ‘population by province’-offset and the ‘year of onset’ as a factor. This ‘year of onset’-factor allowed for the modelling of long-term trends, also including changes in surveillance, and was included to avoid confounding as long-term trends were not eliminated through referent selection.

### Software and code

All analyses were performed in R. The gnm-package was used for conditional Poisson analysis [31]. The DLNM-package was used for the DLNM analysis [25]. A working example of the code was made available at https://zenodo.org/badge/latestdoi/245365464.

## Results

### Descriptive analysis

**Fig. 2.**
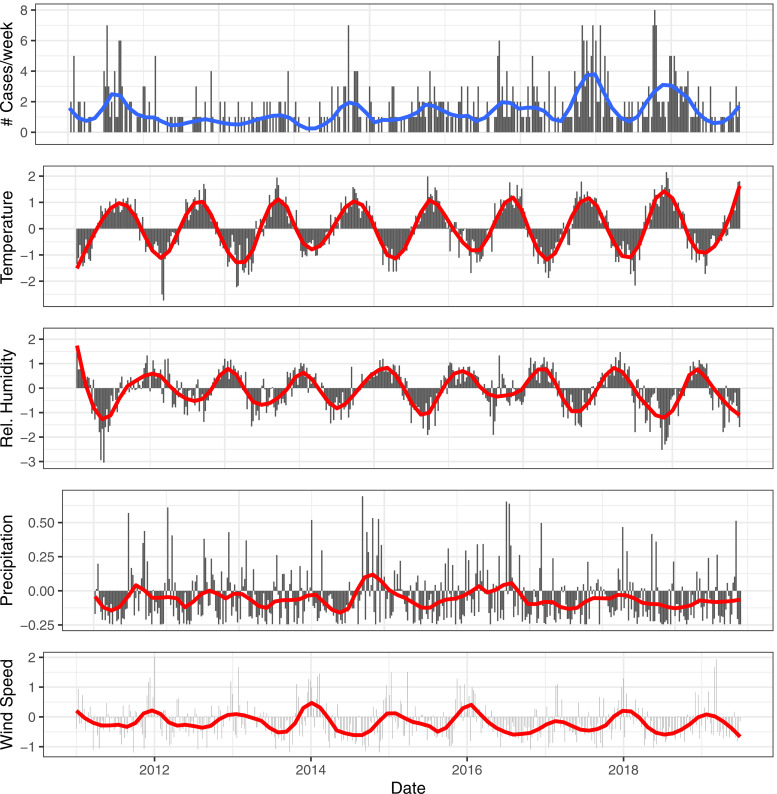
Smoothed (red) and weekly totals (black) for the reported number of cases. Smoothed (red) and scaled meteorological variables (black) for temperature, relative humidity, precipitation and wind speed for the central province Flemish Brabant.

We included 614 cases into the analysis. We observed an overall increase of the reported number of LD cases in Belgium from 55 in 2011 to 78 in 2018. The increase was observed over a seasonal pattern with a yearly peak in August–September (Fig. 2). There were five dates on which three cases were reported and 28 dates on which two cases were reported from the same province. For over 80% of cases, laboratory case confirmation was based on the detection of *Legionella* antigens in urine.

#### Correlation in the exposure and event series

A significant positive linear time trend was detected for the weekly number of cases (estimate on a national level: 0.0026, *P* < 0.001). Not all provinces shared this trend. In Brussels, Namur and Luxembourg provinces, it was not present.

All four meteorological variables had a significant linear time trend, positive for temperature, negative for wind speed, relative humidity and precipitation over the time period. The largest correlations between exposure variables were observed between relative humidity and temperature (Pearson's correlation coefficient = −0.33, *P* < 0.05).

### Case-crossover results

**Fig. 3.**
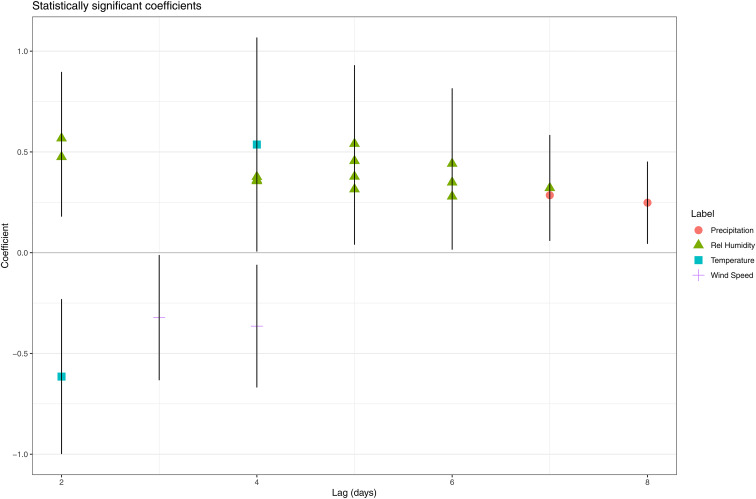
Statistically significant coefficients and their 95% confidence interval obtained by fitting model all days in the ‘at risk’-period separately.

When a model was fitted for each day in the ‘at risk’ period, we observed multiple statistically significant (*P* < 0.05) positive associations between relative humidity and the number of LD cases. For certain days, a higher coefficient was estimated for the higher quantiles compared to the lower quantiles (e.g. 6 days prior to onset) indicating a unidirectional dose–response effect (Table 1). Eight and three days before disease onset were the only lags without a significant association with relative humidity. From the models for day 7 and 8, we obtained a positive significant association between precipitation and the number of LD cases. A negative association was observed for wind speed on day 3 and 4. Both a positive (with the quantile 12.4–16.2 °C on day 4) and negative (with the quantile 4.6–8.5 °C on day 2) association were obtained for temperature (Fig. 3).

**Table 1.** Overview of the significant coefficients when a separate model was fitted for each lag (in number of days before disease onset) (quantile 1 was the reference quantile)

	Lag	Quantile number	Quantile	Estimate	Std. error	*P*-value
Temperature	2	2	4.6–8.5 °C	−0.615	0.196	0.002
Rel. humidity	2	3	78.6–84.4%	0.475	0.151	0.002
Rel. humidity	2	4	84.4–90%	0.568	0.168	0.001
Wind speed	3	4	3.7–4.95 m/s	−0.322	0.159	0.042
Temperature	4	4	12.4–16.2 °C	0.537	0.271	0.048
Rel. humidity	4	2	71.1–78.6%	0.361	0.136	0.008
Rel. humidity	4	3	78.6–84.4%	0.355	0.154	0.021
Rel. humidity	4	4	84.4–90%	0.376	0.173	0.030
Wind speed	4	4	3.7–4.95 m/s	−0.364	0.155	0.019
Rel. humidity	5	2	71.1–78.6%	0.315	0.137	0.021
Rel. humidity	5	3	78.6–84.4%	0.455	0.152	0.003
Rel. humidity	5	4	84.4–90%	0.378	0.172	0.029
Rel. humidity	5	5	90–100%	0.541	0.199	0.007
Rel. humidity	6	2	71.1–78.6%	0.279	0.135	0.038
Rel. humidity	6	4	84.4–90%	0.349	0.168	0.038
Rel. humidity	6	5	90–100%	0.442	0.191	0.021
Rel. humidity	7	2	71.1–78.6%	0.321	0.134	0.017
Precipitation	7	2	>0.2 mm	0.285	0.105	0.006
Precipitation	8	2	>0.2 mm	0.248	0.104	0.017

Std. error = standard error.

Reference quantiles (temperature = −15–4.6 °C; rel. humidity = 20.6–71.1%, wind speed = 0–2.15 (m/s), precipitation ≤0.2 mm).

**Fig. 4.**
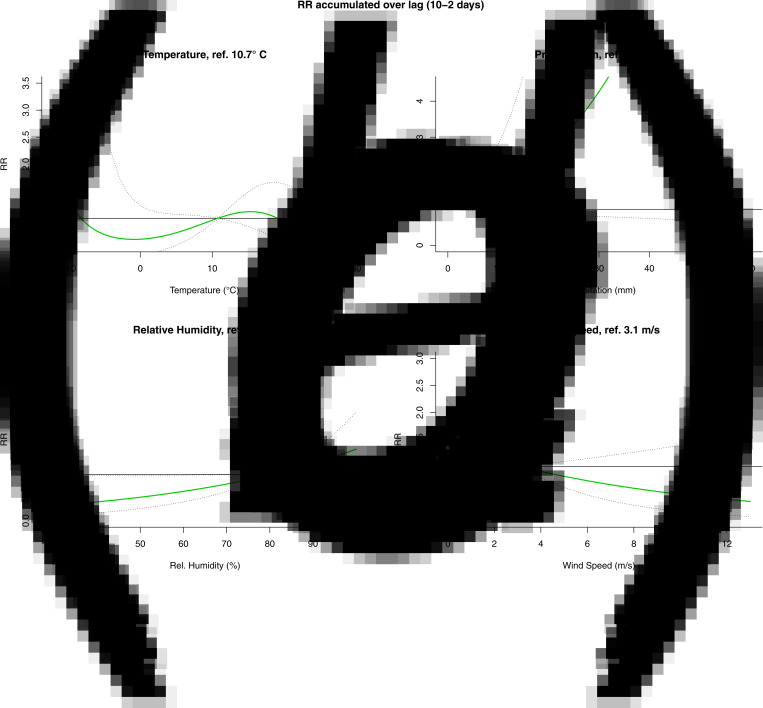
Relative risk (RR) and 95% confidence interval accumulated over the ‘at risk’-period (10 to 2 days prior to disease onset) from the DLNM for temperature (A), precipitation (B), relative humidity (C) and wind speed (D).

**Fig. 5.**
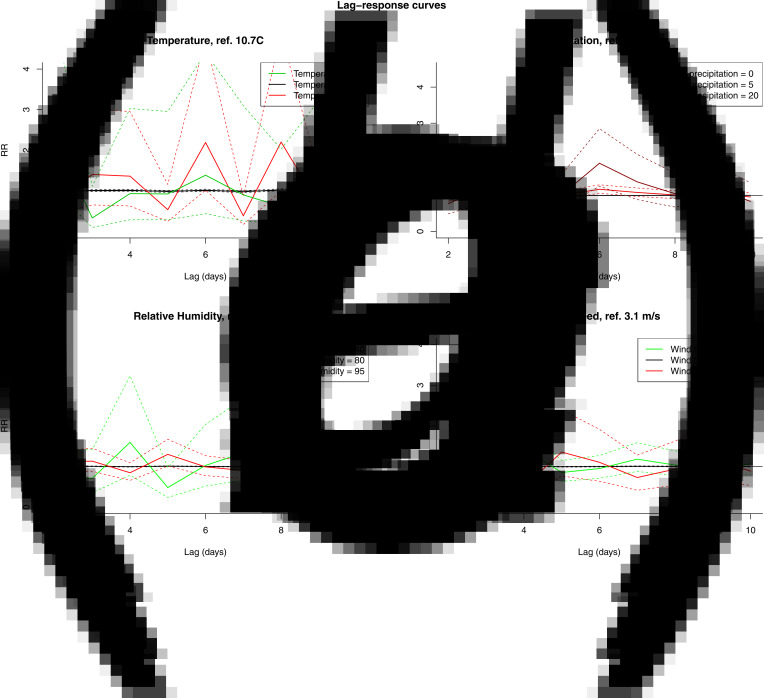
RR by fixed variable values over the days in the ‘at risk’-period (10 to 2 days prior to disease onset) for temperature (A), precipitation (B), relative humidity (C) and wind speed (D).

The results from the DLNM model were presented by accumulating over the lags (Fig. 4) and by selecting two values, mostly one above and one under the reference value, of the meteorological variable (Fig. 5). When accumulating over the ‘at risk’-period, we only observed a significant positive association with relative humidity. When analysing by day in the ‘at risk’-period, positive associations were observed with precipitation on day 6 and relative humidity on day 5. A negative association with wind speed was observed on day 4. There were no significant associations with temperature.

## Discussion

Our main observations were a positive association with relative humidity (5 days before onset with the DLNM, several days when fitting days separately) and a negative association with wind speed (4 days before onset with the DLNM, three and 4 days when fitting days separately). Both methods found positive associations with precipitation, but with a slightly different lag: 6 days for the DLNM, 7 and 8 days when fitting days separately. These main observations are consistent with previously published epidemiological studies and plausible given the biological characteristics of the species. Precipitation can cause *Legionella* to move from its habitat and contaminate surfaces, e.g. transient puddles on asphalt roads. *Legionella* in the puddles can be aerosolised by moving vehicles, increasing the exposure risk [22,32]. Once aerosolised higher relative humidity is associated with longer survival [30]. Some wind speed is necessary for the dissemination of contaminated aerosols. García-Fulgueiras *et al*. reported how an average wind speed of 9 kph gave rise to dispersal of the aerosols during an outbreak implicating a cooling tower [33]. A comparable observation was made by Ferré *et al*. (3.6 kph) [34]. High wind speeds will however result in a decrease of the mean aerosol mass and mean microbial air concentration and disrupt the physical integrity of surface microlayers [35].

Previous epidemiological studies have identified fairly comparable sequences. For example, Fisman and Dunn *et al*. found the largest effects to be higher relative humidity/precipitation on day 9, but they also detected an effect of wind speed on day 7 [5,21]. With respect to temperature, contradictory results have been reported in the direction of the effect (negative [14,15] and positive [16–19]) and in the lag. Halsby *et al*. reported a high disease risk at high temperatures (up to 9 weeks delay) with high relative humidity [17]. Beauté (3 weeks), Brandsema (4 weeks) and Ricketts (10–14 weeks) identified warm weather followed by heavy rainfall as the most favourable conditions for community-acquired LD [6,12,16]. Karagiannis identified warm, humid and showery summer weather as associated with higher LD incidence during the same week [11]. Although we did not investigate the period before incubation, we did observe short-term associations: positive for above average temperatures (quantile 12.4–16.2 °C, day 4) and negative for low temperature (quantile 4.6–8.5 °C, day 2) in the single-day models. We observed no significant association with temperature in the DLNM. Since there were likely issues with the type I error and autocorrelation in the single-day models (see below), a repetition of the DLNM when more statistical power, a longer time series, is present is advised.

### DLNM

In the introduction, we presented three challenges to research on weather effects on LD incidence: non-linearity, seasonality and autocorrelation. To overcome the challenge of autocorrelation we proposed the use of DLNM. We however also used a standard case-crossover design in which several models where fitted for data collected on different days in the ‘at risk’ period. Since DLNMs had not been used previously in case-crossover studies on LD, a comparison to a more standard approach allowed us to better introduce the method.

In general, we observed more significant associations with the single-day models: significant associations with temperature, multiple days with significant associations with wind speed, precipitation and relative humidity. There are two methodological reasons for this observation. (i) Since we did not correct for multiple testing, the probability of a type I error over the complete ‘at risk’-period, nine separate ‘single-day’ models, was higher than 5%. With the DLNM, the probability for a type I error remained at 5%. (ii) There was autocorrelation between consecutive days and between variables. Therefore, if a significant effect was detected at day *x* for a variable, separate models at days *x* − 1 and *x* + 1 were likely to detect significant effects for the same variable but also for correlated variables. Single-day models however also come with the risk of missing significant effects. Consider for example that precipitation increases the change of LD 6 days later, but precipitation is typically followed by above average wind speed, which decreases the risk. Since we generally observe a sequence of effects that cancel each other out, the single-day models will be under powered to detect any of the effects. From a ‘single-day’ point of view, it is hard to detect the increased risk associated with precipitation as precipitation typically is not followed by an increased number of cases. The DLNM however is able to include observations over the entire period when considering the effects at a certain day as it models a trend. As expressed by Karagiannis *et al*. and given the correlations between delay periods and meteorological variables, it makes sense to interpret individual coefficients as parts of weather types and patterns [11]. Given the need to explore sequences and exposures at different delays, we believe that a DLNM is particularly suitable for research on meteorological variables and LD incidence and should be preferred over fitting several single-day models.

Although a DLNM has the advantage of fitting the entire ‘at risk’-period with the same model, additional design choices have to be made on how the lag and covariate effects are to be included in the cross-basis [25]. A piecewise linear model for the lag dimension will estimate a separate coefficient at each of the time points. Alternatively, with a basic cubic spline for the lag dimension, covariate effects will be smoothed over the time points between knots. This is true for covariates with and without temporal autocorrelation. This can be an interesting strategy, especially when the underlying mechanism allows for the accumulation of risk over time. Whenever separate days are to be discussed it might be preferable to include a piecewise linear effect. With LD we can assume that infection will occur on a single day. Risk however might still accumulate over longer time periods. Both modelling choices thus are defendable and this is reflected in previous research as both single days [5,6,22] and values averaged over longer time periods have been used [15,17]. With respect to the covariate dimension of the cross-bases we also opted for linear bases, with the exception of the base for temperature which was a basic spline. Given the array of flexible splines available, this is a conservative choice. Our ‘single-day’ models however did not indicate non-linear effects and previous research has only regularly reported non-linearity (if methods allowed for it) for temperature. Linear effects in a DLNM do however allow for the reference point to differ from zero. Values below and above the reference point have different associations with the outcome. Our choice for relatively simple cross-bases is further motivated by a potential disadvantage of DLNM: they allow for very flexible modelling and, as such, are vulnerable to confounding. This might especially be a concern when high degree splines with multiple knots are used in a context of unmodelled confounding seasonality as it is likely that part of the seasonality will be captured by covariate bases. We have tried to prevent this by using linear bases and the elimination of day-to-day seasonality with a case-crossover design. Future research should investigate the risk of overfitting with DLNM.

### Strengths

The time-stratified referent selection used for the case-crossover study has been discussed by us in a separate paper. In this paper, a simulation study, we reported the elimination of all time-varying confounding with our referent selection strategy given the inclusion of an extra term for long-term trends in the model. The case-crossover model benefits from its underlying intuitive set-up and can easily be combined with a DLNM.

In addition to how the data were fitted, we also tried to improve our analysis during data preparation. Even though Belgium is a small country, we aggregated both case and exposure data by province. Since certain trends were not shared between provinces, this approach has the benefit of less potential confounding. The estimated provincial exposures should also represent the individual exposure better as compared to national estimates.

### Limitations

Seasonality in LD incidence is not investigated in this research as it was eliminated by design. What drives seasonality in LD incidence thus remains unexplored and all our results should be interpreted as ‘out of season’ results. We observed a positive association between relative humidity and LD incidence given seasonality. High relative humidity in itself is not associated to an increase in LD incidence, but high relative humidity in comparison to the corresponding days in the other study years is.

We combined different surveillance sources to include the maximum amount of case information. There was no information on undiagnosed cases and on diagnosed cases not reported to any of the surveillance systems. In this analysis we therefore had to make the assumption that there were no structural differences with respect to the date of onset between reported cases and those not reported or undiagnosed. Our analysis allowed for yearly and provincial differences in the completeness of surveillance and in testing behaviour. Testing behaviour possibly has changed during the study period as the urinary antigen test has been reimbursed from 2016 onwards.

Information on the source of infection was limited and we were only partly able to identify cases with a possibly common source of infection. We could only identify 12 outbreak-related cases. An additional sensitivity analysis from which all outbreak-related cases were removed and one in which they were included, thus could not be performed. An additional reason to include the possible source of infection into the analysis would be to investigate how different sources are affected by meteorology. We aggregated over different *L. pneumophila* genotypes. Different genotypes have different temperature-dependent growth kinetics and serogroups have different survival rates in aerosols. Serogroup 1 is known to survive longer in aerosols than other subtypes [36]. Because the *L. pneumophila* serogroup 1 pathogen likely accounted for over 80% of LD cases, our results mainly reflected the association between meteorological variables and serogroup 1 LD incidence. Differentiating between *L. pneumophila* serogroups and sources seems an interesting topic for future research [37].

## Data Availability

The R code for the analysis and a working example to apply a DLNM to case-crossover designs are available at https://zenodo.org/badge/latestdoi/245365464. The individual case data and data on the meteorological variables cannot be provided due to privacy concerns.
